# Granulomatous Diverticulitis: Who Would Have Thought!

**DOI:** 10.7759/cureus.2795

**Published:** 2018-06-13

**Authors:** Fady G. Haddad, Sandy El Bitar, Hassan Al Moussawi, Liliane Deeb

**Affiliations:** 1 Gastroenterology and Hepatology, Staten Island University Hospital, New York, USA; 2 Internal Medicine, Staten Island University Hospital, New York, USA; 3 Medicine, Staten Island University Hospital, New York, USA

**Keywords:** diverticulosis, segmental diverticulitis, granulomatous, segmental colitis associated with diverticulosis, diverticular disease associated colitis

## Abstract

Diverticular disease (DD) can have different presentations, including chronic colitis. However, diverticular disease-associated colitis (DAC) is a separate entity that can be associated with a granulomatous inflammation. DAC usually affects the left colon with no involvement of the cecum and the ascending colon. In this setting, Crohn's disease is high in the differential diagnosis. Although granulomatous colitis associated with diverticulosis has been previously described, this is the first case affecting the right colon to be reported in the English literature according to our search of the PubMed database. The patient presented with a tumor-like mass abutting the right colon that was further diagnosed as a granulomatous reaction secondary to DAC.

## Introduction

Diverticular disease (DD) encompasses a broad scope of manifestations, including chronic colitis. Diverticular disease-associated colitis (DAC) is a distinct entity that can occasionally exhibit granulomatous inflammation. It usually affects the left colon, excluding the cecum and the ascending colon. In this setting, caution is advised to avoid an inappropriate diagnosis of Crohn's disease. Although granulomatous colitis associated with diverticulosis has been previously described, our patient is the first case affecting the right colon to be reported in the English literature based on our search of the PubMed database. He presented with a hypermetabolic tumor-like mass abutting the right colon that was further identified as a granulomatous reaction secondary to DAC.

## Case presentation

**Figure 1:**
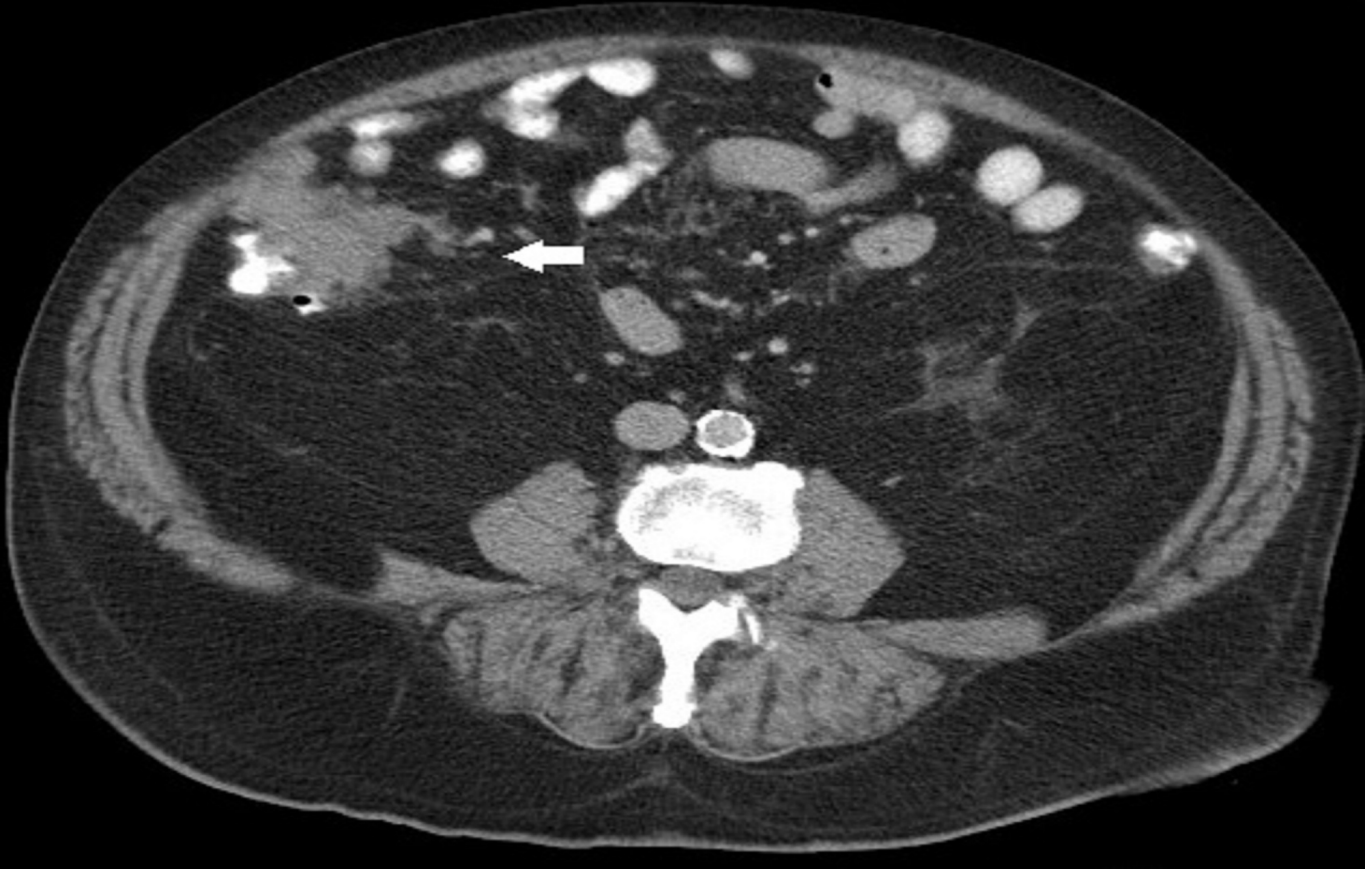
Abdominal computed tomography scan, axial view, showing an irregular 5.5 cm soft tissue mass abutting the right colon medially

**Figure 2:**
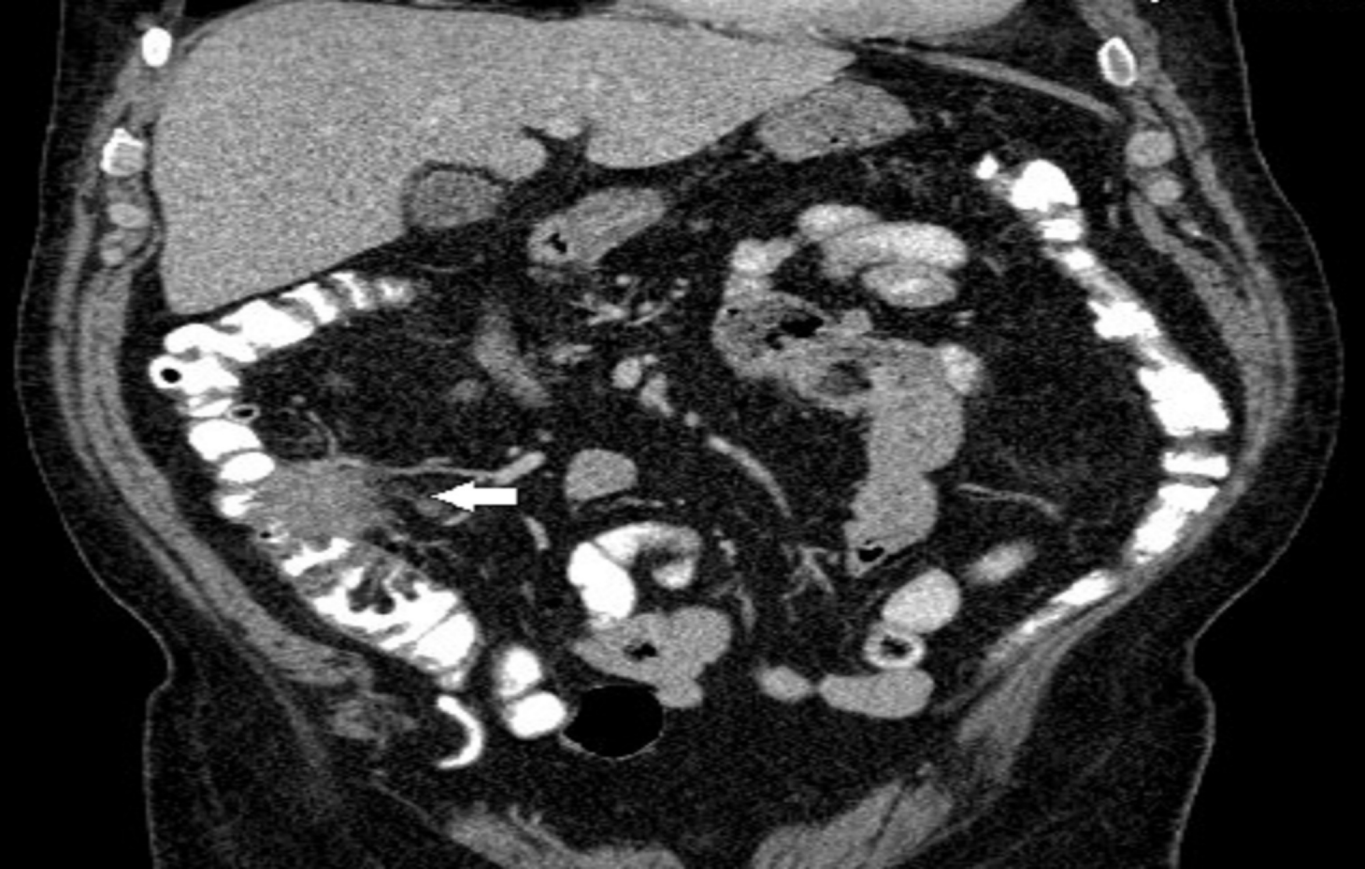
Abdominal computed tomography scan, coronal view, showing an irregular 5.5 cm soft tissue mass abutting the right colon medially

A 77-year-old man with diabetes mellitus and hypertension presented with right lower quadrant (RLQ) abdominal pain that started a few weeks prior to his presentation. Physical examination revealed a palpable RLQ mass. An abdominal computed tomography (CT) scan revealed a 5.5 cm irregular soft tissue mass abutting the ascending colon medially (Figures 1, 2).

**Figure 3:**
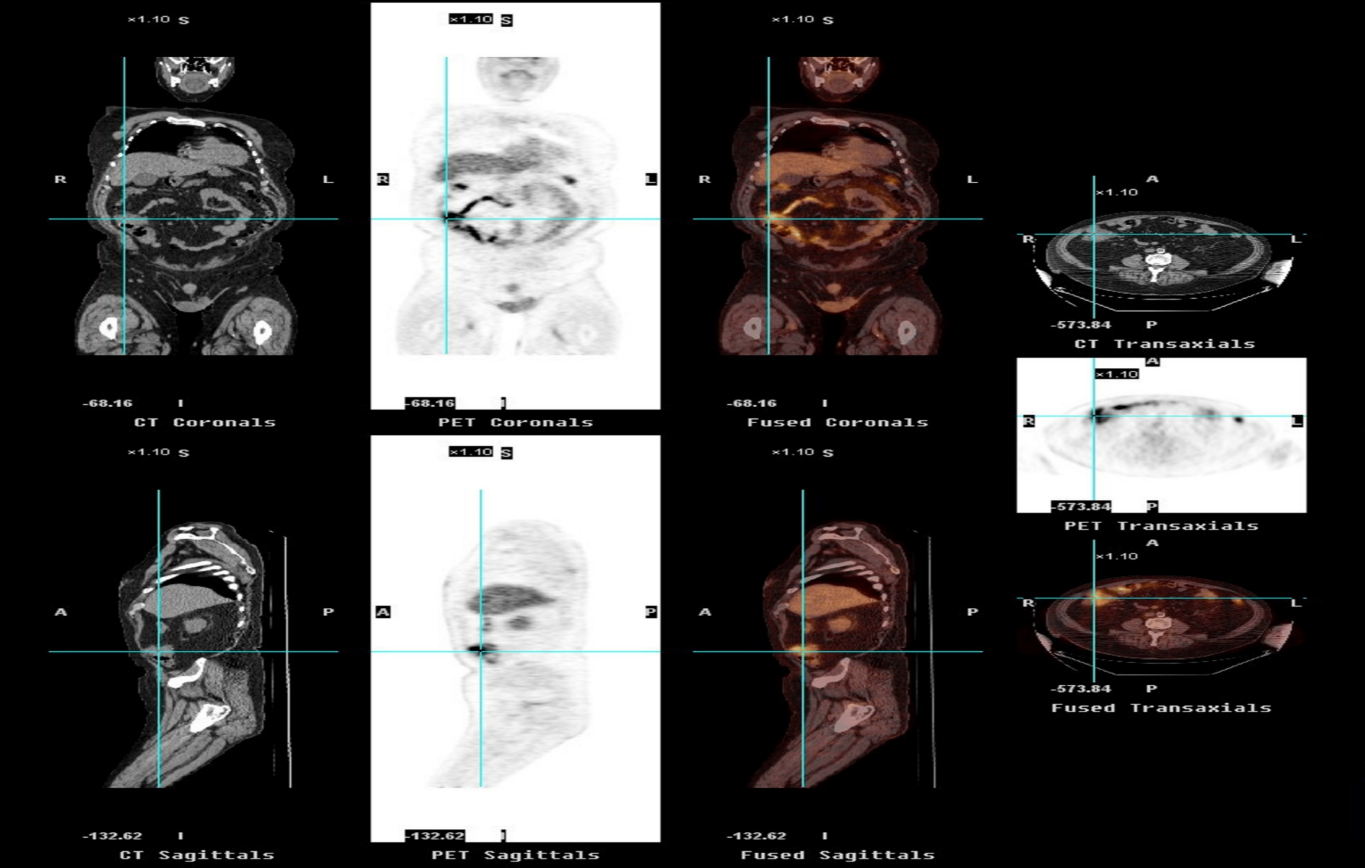
PET scan showing increased metabolic activity of the mass suspicious for biological tumor activity PET: positron emission tomography

Colonoscopy revealed diverticulosis in the sigmoid and ascending colon. On positron emission tomography (PET) scan, the mass exhibited increased metabolic activity suspicious for biological tumor activity (Figure 3).

**Figure 4:**
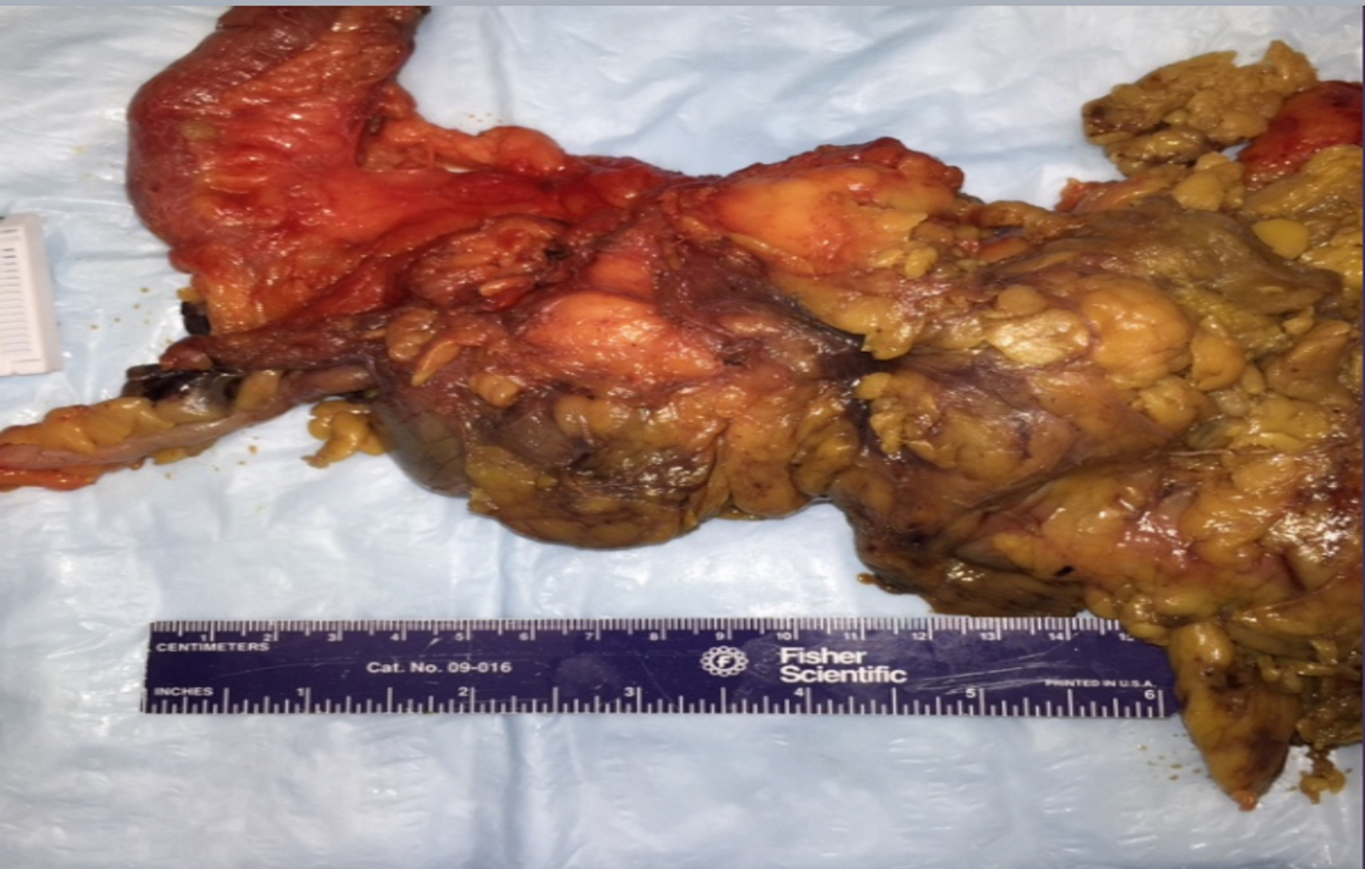
Gross pathology showing diverticulitis with localized suppurative granulomatous inflammation in the pericolic fat

A CT-guided biopsy revealed inflammatory cells. The patient underwent a diagnostic laparoscopy, and a fresh frozen section was inconclusive for malignancy. This was followed by a robotic-assisted right hemicolectomy en bloc with the mass. Pathology showed diverticulitis with localized suppurative granulomatous inflammation in pericolic fat (Figure 4).

**Figure 5:**
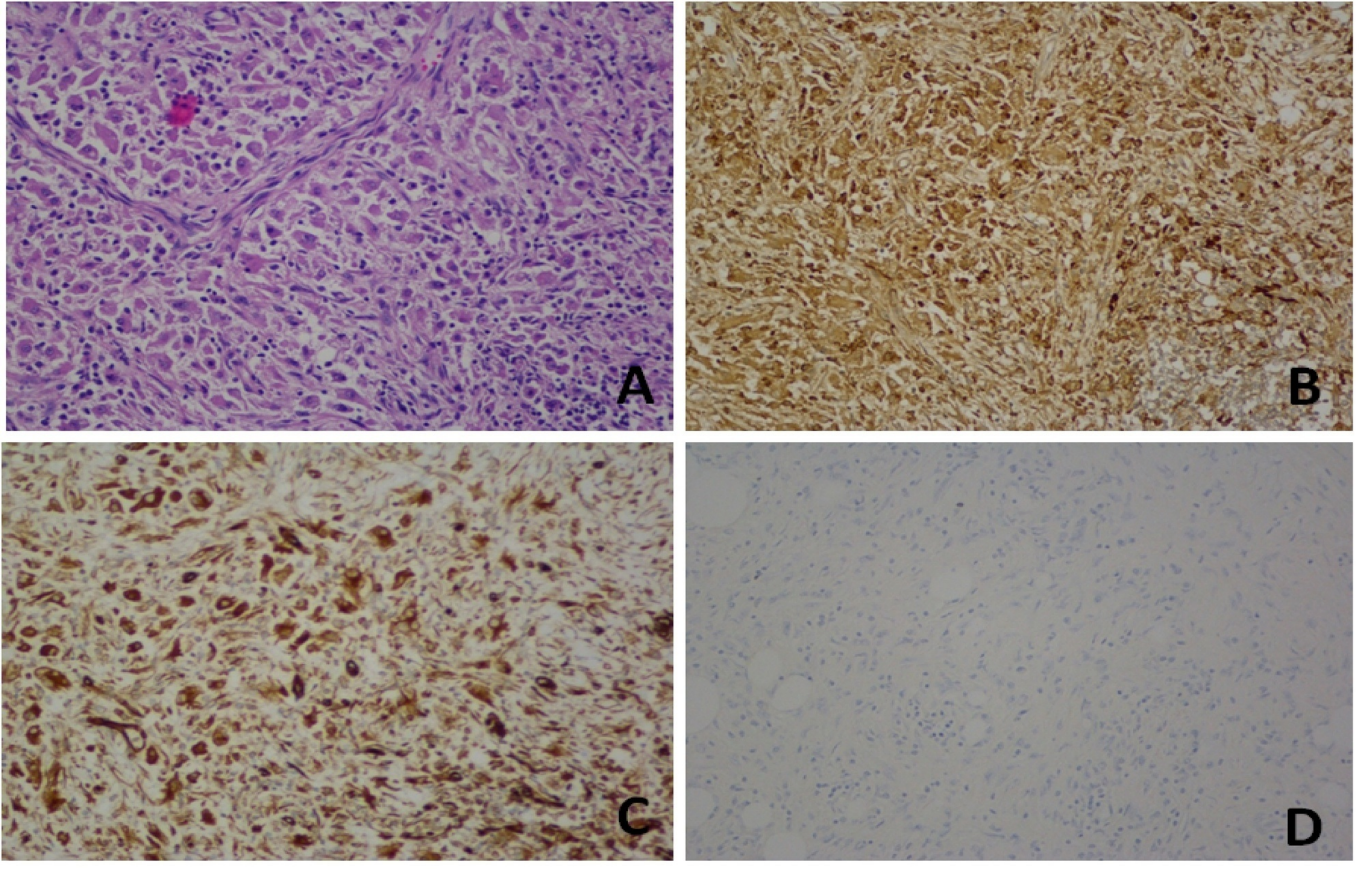
Pathology images Pathology images showing the non-caseating granulomas consisting of epithelioid histiocytes with abundant eosinophilic cytoplasm and eccentric nuclei (A). Immunostains were positive for CD68 (B) and vimentin (C) and negative for pancytokeratin (D).

The non-caseating granulomas consisted of epithelioid histiocytes with abundant eosinophilic cytoplasm and eccentric nuclei (Figure 5A). Immunostains were positive for CD68 (Figure 5B) and vimentin (Figure 5C) and negative for pancytokeratin (Figure 5D), eliminating the possibility of a carcinoma or sarcoma. 

The patient improved clinically and was successfully discharged after few days of his surgery. 

## Discussion

DD is a leading cause of hospitalizations in developed countries affecting 30 - 50% of individuals older than 60 years [1-2]. DAC describes the occurrence of mucosal inflammation in a colon segment affected with DD with relative sparing of the rectum and proximal colon. Its prevalence is suggested to be approximately 1.3 - 3.8% [1-2]. Pathogenesis is multifactorial with multiple reports noting clinicopathological overlap between DAC and inflammatory bowel diseases (IBD), especially in patients with granulomatous colitis, as in our case (Haddad FG, et al.: A Pseudotumor? No! It’s a Granulomatous Diverticulitis in Disguise! American College of Gastroenterology 82nd annual mtg., Orlando, FL, Oct. 13-18, 2017, abstract #1426. http://www.nature.com/articles/ajg2017312.pdf). Recurrence rates and long-term outcomes of DAC are not well-defined and could range from a benign course to an overt IBD. More studies are needed in order to further characterize this entity [1-4].

## Conclusions

Diverticular disease-associated colitis is a known but uncommon presentation of diverticular diseases. A clinical and pathologic overlap between this entity and inflammatory bowel diseases, namely, Crohn’s disease, poses a diagnostic and therapeutic dilemma in the management of affected individuals. In addition, rates of recurrence and long-term outcomes are not well-defined, ranging from a benign course to manifestations of overt IBD. More studies are needed in order to further characterize this entity.
